# Increasing patient-reported allergies are not associated with pain, functional outcomes, or satisfaction following medial patellofemoral ligament reconstruction: a retrospective comparative cohort study

**DOI:** 10.1186/s43019-022-00147-1

**Published:** 2022-04-05

**Authors:** Andrew S. Bi, Dhruv S. Shankar, Kinjal D. Vasavada, Nina D. Fisher, Eric J. Strauss, Michael J. Alaia, Kirk A. Campbell

**Affiliations:** 1 Division of Sports Medicine, NYU Langone Orthopedic Hospital, 333 East 38th Street 4th Floor, New York, NY 10016 USA; 2 NYU Langone Orthopedic Hospital, 301 E 17th St, New York, NY 10003 USA

**Keywords:** Patient-reported allergies, Medial patellofemoral ligament (MPFL), Patient satisfaction, Return to sport

## Abstract

**Background:**

Patient-reported allergies (PRAs) are often stigmatized as a potential nonmodifiable risk factor for increased pain and worse functional outcomes following surgery. However, there is a dearth of literature directly assessing the impact of PRAs on outcomes in sport surgeries such as medial patellofemoral ligament reconstruction (MPFLR). The purpose of our study was to determine whether PRAs were associated with worse outcomes following MPFLR.

**Methods:**

We conducted a retrospective review of patients who underwent MPFLR at our institution from 2011 to 2019. Patients were included if they had at least 12 months of follow-up. PRAs were obtained from preoperative medical assessments and categorized by drug class. Demographic and perioperative data were obtained from electronic medical records. Postoperative outcomes were measured using a telephone survey and included recurrent instability, Visual analog scale (VAS) for pain, VAS for sports, Kujala score, MPFL-Return to Sport after Injury (MPFL-RSI) score, and overall satisfaction score. Multiple linear regression was used to determine association between PRAs and outcome measures, and *p*-values less than 0.05 were considered significant.

**Results:**

The cohort included 141 MPFLR. Most patients were female (98, 70%) with an average age of 25 years (range 12–56 years). Average follow-up time was 47 months. Forty-seven patients (33%) reported at least one PRA. There were no significant differences in postoperative pain, functional outcomes, satisfaction, or return to sport between patients with or without PRAs (all *p* > 0.05). Absence of antibiotic PRAs was predictive of higher VAS (*p* < 0.007), but there were no other differences. There were no significant differences in outcomes between patients without PRAs, PRAs without a concomitant psychiatric disorder, or PRAs with a concomitant psychiatric disorder (all *p* > 0.05).

**Conclusions:**

In conclusion, PRAs with or without concomitant psychiatric diagnoses are not associated with worse postoperative pain, functional outcomes, or satisfaction following MPFLR with allograft, dispelling common misconceptions that increased number of allergies or psychiatric diagnoses lead to inferior surgical outcomes. Presence of antibiotic allergies was associated with lower VAS postoperative pain score. Future research should investigate the relationship between PRAs and other surgeries in the field of sports medicine.

## Introduction

Allergies in the surgical population have been a topic of interest for many years, with most studies focused within the field of anesthesiology, examining validity of patient-reported allergies (PRA) and healthcare providers’ responses to them [1–3]. Whether through anecdotal evidence or scientific literature, orthopedic surgeons often have bias toward patients who report multiple allergies. The arthroplasty literature has demonstrated worse outcomes and higher rates of prosthetic joint infections in patients with increasing PRAs [4–6], as has the spine literature [7, 8]. This is in contrast to shoulder arthroplasty literature, which has reported that PRAs have no association with functional outcomes but higher rates of postoperative pain [9, 10], while foot and ankle literature demonstrated no association between PRAs and patient-reported outcomes [11].

It is important to identify prognostic indicators of worse outcomes following surgery, not to discriminate against certain patients, but to appropriately counsel patients on risks, benefits, and expectations following surgery. While modifiable risk factors such as increased body mass index (BMI) and smoking can have preoperative interventions to decrease risk [12, 13], it is still essential to understand nonmodifiable risk factors such as sex, age, or possibly PRAs [12, 14] to understand reasonable expectations following surgery. In addition, not only have PRAs been stigmatized in association with worse outcomes, but so have concomitant psychiatric diagnoses in a patient’s medical history, with a few studies investigating this relationship [15, 16].

However, there is a dearth of literature examining the relationship between PRAs and sports surgery. To our knowledge, there have been no prior studies evaluating the association between PRAs and functional outcomes following medial patellofemoral ligament reconstruction (MPFLR). The purpose of this study was threefold, to investigate whether (1) the presence or number of allergies had an association with worse functional outcomes, (2) allergies to antibiotics had an association with worse functional outcomes, and (3) allergies and psychiatric diagnoses had any correlation with functional outcomes. Our hypothesis is that patients with preoperative allergies and/or psychiatric diagnoses would correlate with worse functional outcomes following MPFLR.

## Methods

### Study design and cohort selection

We conducted a retrospective review of patients who underwent MPFLR with allograft for treatment of lateral patellar instability at our center from 2011 to 2019. Patients were included in the cohort if they underwent MPFLR with gracilis or semitendinosus allograft and had at least 12 months of follow-up. Surgeries were performed by one of six sports medicine-trained orthopedic surgeons at our center. For the purposes of analysis, patients who underwent bilateral surgeries were treated as two separate subjects for their left- and right-sided procedure.

#### Power analysis and sample size determination

Minimum sample size was estimated using the Kujala Anterior Knee Pain Scale, one of the most common patient-reported outcomes used in the patellar instability literature [17]. Estimated group mean difference between the PRA and no-PRA groups was set at the minimum clinically important difference (MCID) of the Kujala score, which Celik et al. previously determined using an anchor-based method to be 9.5 [18]. Standard deviation of the Kujala score among this cohort was determined to be 15.6 based on a prior analysis [19]. Power analysis was conducted using a desired statistical significance of 0.05 and statistical power of 0.80, and it was determined that a minimum of 44 subjects were required per group.

### Patient-reported allergies

Patient-reported allergies were identified from preoperative medical assessments (POMAs) in each patient’s electronic medical record. Only medication allergies noted in the POMA were included, whereas dietary or seasonal allergies were not included in the analysis. Any allergies that resolved by the time of surgery or developed after the date of surgery were excluded. At our center, drug allergies and specific reactions (ex. urticaria, nausea and vomiting, anaphylaxis) included in POMAs are self-reported by the patient and do not require confirmation by an allergist–immunologist. For each patient, the presence or absence of any PRAs as well as the total number of PRAs were noted. Allergy to at least one antibiotic of any class (ex. beta-lactams, macrolides) was also noted.

In addition to PRAs, prior diagnosis of any diagnosed psychiatric disorder, operative variables such as graft type (gracilis or semitendinosus), tibial tubercle to trochlear groove (TT–TG) distance, and any concomitant procedures were recorded. Postoperative events including reoperations (revision procedure, removal of hardware, etc.) and recurrent patellar instability were also recorded.

### Outcomes measured

Postoperative outcomes were assessed using a telephone survey. Outcomes measured by the survey included presence of recurrent patellar instability, overall pain level as measured by visual analog scale (VAS), pain level during sports and/or physical activity as measured by VAS, anterior knee pain level as measured by Kujala score, psychological readiness to return to sport as measured by MPFL-Return to Sport after Injury (MPFL-RSI) score, overall satisfaction with the procedure on a scale from 0 to 100 (with 100 representing maximum satisfaction), and whether the patient would undergo the same procedure again.

### Statistical methods

All statistics were performed in SAS (version 9.4), and *p*-values less than 0.05 were considered significant. Continuous variables were assessed for normality using the Shapiro–Wilk test. Baseline characteristics were compared between; (1) patients with and without any PRAs, (2) patients with and without antibiotic allergies, (3) patients with no PRAs versus one PRA versus more than one PRA, and (4) patients with no PRAs versus with PRAs and no psychiatric diagnoses versus with PRAs and a psychiatric diagnosis, using Fisher’s exact test for categorical variables and Mann–Whitney *U*-test or Kruskal–Wallis test with Dwass, Steel, Critchlow–Fligner (DSCF) post hoc analysis for continuous variables. If any characteristics were significantly different between groups, they were considered to be confounding variables.

Unadjusted comparisons of outcomes between groups were performed using Fisher’s exact test for binary outcomes (ex. recurrent instability) and Mann–Whitney *U*-test or Kruskal–Wallis test with DSCF post hoc analysis for continuous outcomes (e.g., satisfaction scores). Confounding variable-adjusted comparisons were performed using logistic regression for binary outcomes and multiple linear regression for continuous outcomes.

## Results

### Description of cohort

The cohort included 141 patients; there were 132 unique patients, of whom 9 had bilateral procedures. Most patients were female (98 of 141, 70%) with an average age of 25 years (range 12–56 years). The majority of procedures were performed with gracilis allograft (132 of 141, 94%). Almost half of the cohort (64 of 141, 45%) participated in at least one sport or physical activity prior to surgery. The most common concomitant procedures were tibial tubercle osteotomy (63 of 141, 45%) and chondroplasty (61 of 141, 43%). Reoperation rate was low (14 of 141, 10%), and the most common reoperation was manipulation under anesthesia (6 of 141, 4%). Average follow-up time was 47 months (standard deviation 28 months).

### Presence of patient-reported allergies versus outcomes

Forty-seven patients (33%) reported at least one PRA. Allergies to antibiotics were most prevalent (32 subjects, 23%), followed by opioids (9 subjects, 6%) and nonsteroidal antiinflammatory drugs (NSAIDs) (6 subjects, 4%). Patients reporting at least one PRA were significantly older than patients reporting no PRAs (*p* = 0.02), but there were no other significant differences in baseline characteristics (Table 1). Unadjusted comparison of outcomes between the groups found no significant differences in incidence of recurrent instability (*p* = 0.79), VAS (*p* = 0.23), VAS for sports (*p* = 0.45), Kujala score (*p* = 0.51), MPFL-RSI score (*p* = 0.86), overall satisfaction (*p* = 0.77), willingness to repeat surgery (*p* = 0.81), RTS (*p* = 0.61), RTS at the presymptomatic level (*p* = 0.40), or time to RTS (*p* = 0.49). After adjusting for age, there were still no significant differences in outcomes between the two groups in terms of recurrent instability (*p* = 0.97), VAS (*p* = 0.21), VAS for sports (*p* = 0.26), Kujala score (*p* = 0.94), MPFL-RSI score (*p* = 0.53), overall satisfaction (*p* = 0.54), willingness to repeat surgery (*p* = 0.60), RTS (*p* = 0.80), RTS at the presymptomatic level (*p* = 0.30), or time to RTS (*p* = 0.48).

**Table 1:** Demographics, operative characteristics, and postoperative outcomes of patients with reported drug allergies (PRAs) versus those without

	All subjects (*n* = 141)	No PRAs (*n* = 94)	With PRAs (*n* = 47)	*p*-Value
Age at time of surgery (years)	25 ± 9	24 ± 9	28 ± 10	0.01*
Sex				
Male	43 (30%)	31 (33%)	12 (26%)	0.37
Female	98 (70%)	63 (67%)	35 (74%)	
Sports participation	64 (45%)	42 (45%)	22 (47%)	0.81
Concomitant procedures				
Primary TTO	64 (45%)	43 (46%)	21 (45%)	0.90
Meniscectomy	5 (4%)	3 (3%)	2 (4%)	1.00
Chondroplasty	61 (43%)	45 (48%)	17 (36%)	0.35
Loose body removal	15 (11%)	10 (11%)	5 (11%)	1.00
Subsequent surgery				
Any reoperation	14 (10%)	7 (7%)	7 (15%)	0.23
Revision	3 (2%)	2 (2%)	1 (2%)	1.00
Removal of hardware	3 (2%)	2 (2%)	1 (2%)	1.00
MUA	5 (4%)	2 (2%)	3 (6%)	0.33
Secondary TTO	2 (1%)	1 (1%)	1 (2%)	1.00
TKA	1 (1%)	0 (0%)	1 (2%)	0.33
Follow-up time (months)	47 ± 28	45 ± 27	51 ± 28	0.22
Outcomes				
Recurrent instability	17 (12%)	11 (12%)	6 (13%)	0.82
VAS (overall)	14 ± 20	15 ± 21	12 ± 19	0.23
VAS (sports)	27 ± 27	28 ± 28	24 ± 26	0.45
Kujala score	85 ± 16	85 ± 16	84 ± 16	0.51
MPFL-RSI score	60 ± 27	59 ± 27	61 ± 28	0.86
Satisfaction score	84 ± 26	83 ± 27	85 ± 24	0.78
Would repeat surgery	117 (83%)	77 (82%)	40 (85%)	0.63
Return to sport	29 (45%)	20 (48%)	9 (41%)	0.61
Return to sport at presymptomatic level	20 (31%)	15 (36%)	5 (23%)	0.40
Time to return to sport (months)	9 ± 6	9 ± 5	11 ± 7	0.49

Results reported as *n* (%) or mean ± standard deviation (SD)

*BMI* body mass index, *TTO* tibial tubercle osteotomy, *MUA* manipulation under anesthesia, *TKA* total knee arthroplasty, *VAS* visual analog scale, *MPFL-RSI* Medial Patellofemoral Ligament-Return to Sport after Injury

*Indicates statistically significant values

### Presence of antibiotic allergies versus outcomes

Thirty-two patients (23%) reported at least one antibiotic allergy, most commonly to penicillin (17 patients, 12%). There were no significant differences in baseline characteristics between patients reporting at least one antibiotic allergy versus those reporting no antibiotic allergies (Table 2). Unadjusted comparison of outcomes between the groups found significantly higher VAS among patients without antibiotic allergies (*p* = 0.007) but no differences in incidence of recurrent instability (*p* = 0.36), VAS for sports (*p* = 0.13), Kujala score (*p* = 0.66), MPFL-RSI score (*p* = 0.79), overall satisfaction (*p* = 0.40), willingness to repeat surgery (*p* = 0.60), RTS (*p* = 0.69), RTS at the presymptomatic level (*p* = 1.00), or time to RTS (*p* = 0.66). Adjusted comparisons were not performed due to absence of confounding variables.

**Table 2:** Demographics, operative characteristics, and postoperative outcomes of subjects with antibiotic allergies versus those without

	No antibiotic PRAs (*n* = 109)	With antibiotic PRAs (*n* = 32)	*p*-Value
Age at time of surgery (years)	25 ± 10	25 ± 7	0.29
Sex			
Male	31 (28%)	12 (38%)	0.33
Female	78 (72%)	20 (62%)	
Sports participation	47 (43%)	17 (53%)	0.28
Concomitant procedures			
Primary TTO	51 (47%)	13 (41%)	0.55
Meniscectomy	5 (5%)	0 (0%)	0.59
Chondroplasty	48 (44%)	13 (41%)	1.00
Loose body removal	12 (11%)	3 (9%)	1.00
Subsequent surgery			
Any reoperation	11 (10%)	3 (9%)	1.00
Revision	3 (3%)	0 (0%)	1.00
Removal of hardware	2 (2%)	1 (3%)	0.54
MUA	4 (4%)	1 (3%)	1.00
Secondary TTO	2 (2%)	0 (0%)	1.00
TKA	0 (0%)	1 (3%)	0.23
Follow-up time (months)	46 ± 29	51 ± 24	0.21
Outcomes			
Recurrent instability	15 (14%)	2 (6%)	0.36
VAS (overall)	16 ± 21	7 ± 15	0.007*
VAS (sports)	29 ± 28	21 ± 25	0.13
Kujala score	84 ± 16	86 ± 15	0.66
MPFL-RSI score	59 ± 27	61 ± 28	0.79
Satisfaction score	83 ± 26	85 ± 26	0.40
Would repeat surgery	89 (82%)	28 (88%)	0.44
Return to sport	22 (47%)	7 (41%)	0.69
Return to sport at presymptomatic level	15 (32%)	5 (29%)	1.00
Time to return to sport (months)	9 ± 5	11 ± 8	0.66

Results reported as *n* (%) or mean ± standard deviation (SD)

*BMI* body mass index, *TTO* tibial tubercle osteotomy, *MUA* manipulation under anesthesia, *TKA* total knee arthroplasty, *VAS* visual analog scale, *MPFL-RSI* Medial Patellofemoral Ligament-Return to Sport after Injury

*Indicates statistically significant values

### Number of patient-reported allergies versus outcomes

Ninety-four patients (67%) reported no PRAs, 22 (16%) reported one PRA, and 10 (7%) reported more than one PRA. Patients reporting more than one PRA were significantly older than patients reporting no PRAs (*p* = 0.03), but there were no other significant differences in baseline characteristics between the three groups (Table 3). Unadjusted comparison of outcomes between the groups found no significant differences in incidence of recurrent instability (*p* = 0.43), VAS (*p* = 0.47), VAS for sports (*p* = 0.51), Kujala score (*p* = 0.94), MPFL-RSI score (*p* = 0.77), overall satisfaction (*p* = 0.96), willingness to repeat surgery (*p* = 0.69), RTS (*p* = 0.74), RTS at the presymptomatic level (*p* = 0.32), or time to RTS (*p* = 0.17). After adjusting for age, there were still no significant differences in outcomes between the three groups in terms of recurrent instability (*p* = 0.50), VAS (*p* = 0.46), VAS for sports (*p* = 0.31), Kujala score (*p* = 0.94), MPFL-RSI score (*p* = 0.64), overall satisfaction (*p* = 0.70), willingness to repeat surgery (*p* = 0.59), RTS (*p* = 0.89), RTS at the presymptomatic level (*p* = 0.88), or time to RTS (*p* = 0.58).

**Table 3:** Patients without patient-reported drug allergies (PRAs) versus those with one versus patients with more than one

	No PRAs (*n* = 94)	1 PRA (*n* = 33)	> 1 PRA (*n* = 14)	*p*-Value
Age at time of surgery (years)	24 ± 9	27 ± 11	29 ± 6	0.02*
Sex				
Male	31 (33%)	9 (27%)	3 (21%)	0.61
Female	63 (67%)	24 (73%)	11 (79%)	
Sports participation	42 (45%)	16 (48%)	6 (43%)	0.88
Concomitant procedures				
Primary TTO	43 (46%)	16 (48%)	5 (36%)	0.72
Meniscectomy	3 (3%)	2 (6%)	0 (0%)	0.77
Chondroplasty	45 (48%)	10 (30%)	7 (50%)	0.38
Loose body removal	10 (11%)	4 (12%)	1 (7%)	0.91
Subsequent surgery				
Any reoperation	7 (7%)	4 (12%)	3 (21%)	0.16
Revision	2 (2%)	1 (3%)	0 (0%)	1.00
Removal of hardware	2 (2%)	0 (0%)	1 (7%)	0.39
MUA	2 (2%)	2 (6%)	1 (7%)	0.23
Secondary TTO	1 (1%)	1 (3%)	0 (0%)	0.56
TKA	0 (0%)	0 (0%)	1 (7%)	0.10
Follow-up time (months)	45 ± 27	52 ± 30	49 ± 23	0.37
Outcomes				
Recurrent instability	11 (12%)	3 (9%)	3 (21%)	0.43
VAS (overall)	15 ± 21	12 ± 19	11 ± 19	0.47
VAS (sports)	28 ± 28	21 ± 25	31 ± 30	0.51
Kujala score	85 ± 16	85 ± 15	83 ± 17	0.80
MPFL-RSI score	59 ± 27	63 ± 28	56 ± 29	0.77
Satisfaction score	83 ± 27	86 ± 19	81 ± 34	0.96
Would repeat surgery	77 (82%)	27 (82%)	13 (93%)	0.58
Return to sport	20 (48%)	6 (38%)	3 (50%)	0.74
Return to sport at presymptomatic level	15 (36%)	4 (25%)	1 (17%)	0.32
Time to return to sport (months)	9 ± 5	9 ± 8	14 ± 2	0.17

Results reported as *n* (%) or mean ± standard deviation (SD)

*BMI* body mass index, *TTO* tibial tubercle osteotomy, *MUA* manipulation under anesthesia, *TKA* total knee arthroplasty, *VAS* visual analog scale, *MPFL-RSI* Medial Patellofemoral Ligament-Return to Sport after Injury

*Indicates statistically significant values

### Patient-reported allergies with concomitant psychiatric disorder versus outcomes

Forty-seven patients (33%) had a diagnosis of a psychiatric disorder, of whom 12 (9%) also reported at least one drug allergy (Table 4). The most common psychiatric diagnoses were anxiety disorders (29 patients, 21%), depressive disorders (22 patients, 16%), and attention-deficit hyperactivity disorder (8 patients, 6%). There were significant differences in age between patients without PRAs, patients with PRAs without psychiatric disorders, and patients with PRAs and psychiatric disorders (*p* = 0.04), but there were no other significant differences in baseline characteristics between the three groups. Unadjusted comparison of outcomes between the groups found no significant differences in incidence of recurrent instability (*p* = 0.25), VAS (*p* = 0.30), VAS for sports (*p* = 0.31), Kujala score (*p* = 0.67), MPFL-RSI score (*p* = 0.35), overall satisfaction (*p* = 0.47), willingness to repeat surgery (*p* = 0.13), RTS (*p* = 0.86), RTS at the presymptomatic level (*p* = 0.15), or time to RTS (*p* = 0.74). After adjusting for age, there were still no significant differences in outcomes between the three groups in terms of recurrent instability (*p* = 0.27), VAS (*p* = 0.42), VAS for sports (*p* = 0.20), Kujala score (*p* = 0.31), MPFL-RSI score (*p* = 0.31), overall satisfaction (*p* = 0.28), willingness to repeat surgery (*p* = 0.14), RTS (*p* = 0.85), RTS at the presymptomatic level (*p* = 0.99), or time to RTS (*p* = 0.78).

**Table 4:** Analysis of MPFL outcomes between patients with allergies with and without psychiatric diagnoses

	No PRAs (*n* = 94)	PRAs without psychiatric disorder (*n* = 35)	PRAs with psychiatric disorder (*n* = 12)	*p*-Value
Age at time of surgery (years)	24 ± 9	28 ± 11	26 ± 5	0.04*
Sex				
Male	31 (33%)	10 (29%)	2 (17%)	0.49
Female	63 (67%)	25 (71%)	10 (83%)	
Sports participation	42 (45%)	16 (46%)	6 (50%)	1.00
Concomitant procedures				
Primary TTO	43 (46%)	15 (43%)	6 (50%)	0.91
Meniscectomy	3 (3%)	2 (6%)	0 (0%)	0.75
Chondroplasty	45 (48%)	13 (37%)	4 (33%)	0.66
Loose body removal	10 (11%)	5 (14%)	0 (0%)	0.46
Subsequent surgery				
Any reoperation	7 (7%)	5 (14%)	2 (17%)	0.30
Revision	2 (2%)	0 (0%)	1 (8%)	0.25
Removal of hardware	2 (2%)	1 (3%)	0 (0%)	1.00
MUA	2 (2%)	3 (9%)	0 (0%)	0.15
Secondary TTO	1 (1%)	1 (3%)	0 (0%)	0.33
TKA	0 (0%)	0 (0%)	1 (8%)	0.09
Follow-up time (months)	45 ± 27	52 ± 26	49 ± 34	0.29
Outcomes				
Recurrent instability	11 (12%)	3 (9%)	3 (25%)	0.25
VAS (overall)	15 ± 21	13 ± 19	9 ± 19	0.30
VAS (sports)	28 ± 28	21 ± 25	33 ± 31	0.31
Kujala score	85 ± 16	86 ± 13	79 ± 20	0.67
MPFL-RSI score	59 ± 27	64 ± 28	52 ± 29	0.35
Satisfaction score	83 ± 27	88 ± 21	76 ± 31	0.47
Would repeat surgery	77 (82%)	32 (91%)	8 (67%)	0.13
Return to sport	20 (48%)	7 (44%)	2 (33%)	0.86
Return to sport at presymptomatic level	15 (36%)	5 (31%)	0 (0%)	0.15
Time to return to sport (months)	9 ± 5	11 ± 8	11 ± 6	0.74

Results reported as *n* (%) or mean ± standard deviation (SD)

*BMI* body mass index, *TTO* tibial tubercle osteotomy, *MUA* manipulation under anesthesia, *TKA* total knee arthroplasty, *VAS* visual analog scale, *MPFL-RSI* Medial Patellofemoral Ligament-Return to Sport after Injury

*Indicates statistically significant values

## Discussion

Contrary to our hypothesis, our analysis found no significant association between PRAs and postoperative pain, patient-reported outcomes, satisfaction, or return to sport following MPFLR. Patients with PRAs had equivalent outcomes to their counterparts without, and an increasing number of PRAs was not associated with worse outcomes. However, patients without an antibiotic allergy were found to have higher postoperative VAS than patients with an antibiotic allergy, though no other differences in outcomes were observed.

Patient-reported drug allergies have a direct impact on perioperative planning of orthopedic surgeries, often necessitating substitutions of anesthetics, intraoperative antibiotics, and postoperative pain medications to prevent adverse drug reactions. As a result, a growing body of literature has explored the potential effects of self-reported drug allergies on surgical outcomes. A few studies have provided limited evidence that allergies may be associated with worse outcomes following total hip arthroplasty (THA) or total knee arthroplasty (TKA). Otero et al. reported lower SF-36 and WOMAC scores among patients with allergies in a prospective cohort of THA and TKA patients [20]. Fisher et al. conducted a retrospective analysis that found that THA and TKA patients with a higher number of PRAs had a higher risk for prosthetic joint infection (PJI) [6]. Similar negative associations between PRAs and outcomes have been found for other orthopedic procedures such as spine surgery and hip arthroscopy [7, 21].

However, these results have not been consistently demonstrated, with several studies correlating our findings in other orthopedic procedures. Nixon et al. performed a retrospective review of 159 patients who underwent elective foot and ankle surgery, and found equivalent outcomes preoperatively, postoperatively, and in the change in patients with PRAs and those without PRAs using the Patient-Reported Outcome Measurement Information System (PROMIS) [11]. They believed that the prior arthroplasty literature demonstrating an association between PRAs and worse outcomes, while showing statistically significant difference, did not demonstrate clinically significant differences. Coxe et al. found that the presence of allergies did not affect levels of postoperative narcotic usage, pain, or satisfaction following elective hand surgery [22]. This discrepancy between studies may be due to the heterogeneity of the populations studied as well as differences in the specific outcomes measured.

Even within the same procedure, the literature is conflicted. Kennon et al. and Rosenthal et al. reported no differences in functional outcomes among shoulder arthroplasty patients with self-reported metal allergies or increasing PRAs in cohorts of 52 and 98 arthroplasties, respectively [9, 23]. However, a retrospective review of 415 shoulder arthroplasties by Menendez et al. found PRAs to be the strongest predictor of postoperative pain, greater than preoperative opioid use, lower American Shoulder and Elbow Surgeons score, and depression [10]. In contrast to most prior research on this topic, our study focused on a young, athletic patient population with few comorbidities, and to our knowledge is the first to specifically assess the association of PRAs with pain, functional outcomes, or satisfaction following MPFLR. MPFLR with allograft presents a unique cohort of patients when compared with knee, hip, or shoulder arthroplasty patients, as the use of allograft adds another variable. It was our initial thought that perhaps patients with increasing number of allergies would have an underlying “global hypersensitivity” and perhaps be more prone to graft rejection [24], but we did not find any significant differences in revision surgery, pain, or functional outcomes between groups.

A significant secondary finding of our study was that, when investigating PRAs to antibiotics, patients *without* antibiotic allergies actually had higher postoperative VAS pain scores at final follow-up than patients with antibiotic allergies. Antibiotic allergies in TKA and THA literature have been of interest due to increased PJI rates when alternative antibiotic prophylaxis is used due to PRAs to penicillins or cephalosporins [25, 26].

Our findings and prior literature beg the question of why PRAs have become associated, whether scientifically or anecdotally, with worse outcomes across multiple specialties. A prospective study of 209 patients by Ferrer et al. sought to examine other confounders between PRAs and postoperative outcomes, specifically psychiatric factors [15]. At preoperative assessments, patients filled out assessments for both PRAs and psychiatric tests for anxiety, as well as pre- and postoperative WOMAC, SF-12, and KSS scores. They found that presence of anxiety was more prevalent in patients with PRAs and that patients with PRAs had worse functional outcomes than those without, but that when controlling for anxiety, there were no significant differences in outcomes between patients with or without PRAs. The same group who found no differences in outcomes in elective foot and ankle surgery between patients with and without PRAs found that preoperative anxiety based on PROMIS anxiety scores predicted worse postoperative pain and functional outcomes [16]. There is a well-established correlation between anxiety and allergic conditions such as atopic dermatitis in the dermatology literature [27, 28], which may be linked to anxiety-provoked immune hypersensitivity [29], but in our analysis, we found no associations between psychiatric diagnoses, PRAs, and postoperative VAS pain levels, functional outcomes, or satisfaction.

## Limitations

There are several limitations to the present study. Due to its retrospective design and low sample size resulting in an underpowered study, some differences may not have been detected. Given that we examined solely patients who had undergone MPFLR, the external validity of our study for other sports surgeries may be limited. We relied on our institution’s protocol of recording self-reporting of allergies by patients to document in the electronic medical record, contributing to potential recall bias. Lastly, only postoperative outcome scores as opposed to change in outcome scores were measured due to the retrospective nature of the study.

## Conclusion

Patient-reported allergies with or without concomitant psychiatric diagnoses are not associated with worse postoperative pain, functional outcomes, or satisfaction following MPFL reconstruction with allograft, dispelling common misconceptions that patients with increased number of allergies or psychiatric diagnoses lead to inferior surgical outcomes. Presence of antibiotic allergies was associated with lower VAS postoperative pain score. Future research should investigate the relationship between PRAs and other surgeries in the field of sports medicine.

## Data Availability

The datasets used and/or analyzed during the current study are available from the corresponding author on reasonable request.
